# Chronic Hypoxia Increases TRPC6 Expression and Basal Intracellular Ca^2+^ Concentration in Rat Distal Pulmonary Venous Smooth Muscle

**DOI:** 10.1371/journal.pone.0112007

**Published:** 2014-11-03

**Authors:** Lei Xu, Yuqin Chen, Kai Yang, Yingfeng Wang, Lichun Tian, Jie Zhang, Elizabeth Wenqian Wang, Dejun Sun, Wenju Lu, Jian Wang

**Affiliations:** 1 Guangzhou Institute of Respiratory Diseases, State Key Laboratory of Respiratory Diseases, The First Affiliated Hospital of Guangzhou Medical University, Guangzhou, Guangdong, China; 2 Division of Pulmonary and Critical Care Medicine, School of Medicine, Johns Hopkins University, Baltimore, Maryland, United States of America; 3 Division of Pulmonary and Critical Care Medicine, Inner Mongolia People's Hospital, Huhhot, Inner Mongolia, China; 4 Geisinger Medical Center, Danville, Pennsylvania, United States of America; University of Illinois College of Medicine, United States of America

## Abstract

**Background:**

Hypoxia causes remodeling and contractile responses in both pulmonary artery (PA) and pulmonary vein (PV). Here we explore the effect of hypoxia on PV and pulmonary venous smooth muscle cells (PVSMCs).

**Methods:**

Chronic hypoxic pulmonary hypertension (CHPH) model was established by exposing rats to 10% O_2_ for 21 days. Rat distal PVSMCs were isolated and cultured for *in vitro* experiments. The fura-2 based fluorescence calcium imaging was used to measure the basal intracellular Ca^2+^ concentration ([Ca^2+^]_i_) and store-operated Ca^2+^ entry (SOCE). Quantitative RT-PCR and western blotting were performed to measure the expression of mRNA and levels of canonical transient receptor potential (TRPC) protein respectively.

**Results:**

Hypoxia increased the basal [Ca^2+^]_i_ and SOCE in both freshly dissociated and serum cultured distal PVSMCs. Moreover, hypoxia increased TRPC6 expression at mRNA and protein levels in both cultured PVSMCs exposed to prolonged hypoxia (4% O_2_, 60 h) and distal PV isolated from CHPH rats. Hypoxia also enhanced proliferation and migration of rat distal PVSMCs.

**Conclusions:**

Hypoxia induces elevation of SOCE in distal PVSMCs, leading to enhancement of basal [Ca^2+^]_i_ in PVSMCs. This enhancement is potentially correlated with the increased expression of TRPC6. Hypoxia triggered intracellular calcium contributes to promoted proliferation and migration of PVSMCs.

## Introduction

Pulmonary hypertension may occur either as a primary disease or as a complication of some pulmonary disorders such as chronic obstructive pulmonary disease (COPD), and progressively leads to heart failure and increased mortality. In the pulmonary vasculature, exposure to hypoxia is associated with vasoconstriction and vasculature remodeling which contributes to pulmonary hypertension [1]. Calcium entry from the extracellular space is a critical step during the hypoxia induced pulmonary vascular smooth muscle contraction. The process involves store-operated calcium channels [2], which have been indicated to be composed of the mammalian homologs of transient receptor potential canonical (TRPC) proteins [3], [4]. To date, seven members of the TRPC family have been identified in mammals (TRPC1-7). Amongst them, TRPC1, TRPC4, and TRPC6 have been demonstrated to be present in rat pulmonary vasculature and are associated with increased store-operated calcium entry (SOCE), elevated basal intracellular Ca^2+^ concentration ([Ca^2+^]_i_) and the proliferation and migration of PASMCs [5], [6]. TRPC1 and TRPC6 have been demonstrated to be up-regulated by hypoxia and are associated with the SOCE enhancement in PASMCs [7].

Pulmonary vessels consist of three longitudinal vascular segments: the arteries, microvessels, and veins. In earlier studies, pulmonary arteries attracted much attention while pulmonary veins (PV) were seen simply as conduit vessels causing them to be largely overlooked. Over the past few decades, increasing evidence indicates important role of PV in regulation of pulmonary circulation [8]. Pulmonary venous system plays an indispensable role in regulating both distention and recruitment of blood flow from alveolar wall capillaries, and thus facilitates the ventilation-perfusion matching in the lungs. Studies in a variety of species indicate that pulmonary veins can exhibit vascular contraction in response to a number of vasoconstrictor stimuli including hypoxia [9]. During hypoxic exposure, vasoconstriction and structural alterations occur both in PA and PV, both contributing a significant portion to total pulmonary vascular resistance [9], [10], [11], [12], [13], [14], [15]. Therefore, to explore the mechanism underlying the development of hypoxic pulmonary hypertension from the sight of PV is of great importance.

It was reported that hypoxia could lead to contraction and remodeling of the pulmonary vein. However, the underlying mechanism remains unclear. Our recent report described the occurrence of store-operated Ca^2+^ entry (SOCE) and presence of TRPC1, 6 in rat distal Pulmonary vein smooth muscles (PVSMCs) [16]. Moreover, we found that acute hypoxia (4% O_2_) can elevates basal [Ca^2+^]_i_ and triggers SOCE in PVSMCs [17], however, whether chronic hypoxia also enhances basal [Ca^2+^]_i_ and SOCE is still unknown. Therefore, in this study, we aim to determine whether chronic hypoxia affects the SOCE and TRPCs expression in rat distal PV, as well as the proliferation and migration of PVSMCs. Our work could enrich our understanding of the mechanism underlying the hypoxic pulmonary vein contraction and remodeling.

## Methods

### PVSMCs isolation and culture

The Animal experiment protocols were approved by the Animal Care and Use Committee of Guangzhou Medicine University and the Animal Care and Use Committee of the Johns Hopkins Medical Institution. All surgery was performed under anesthesia with sodium pentobarbital (65 mg/kg i.p.), and all efforts were made to minimize animal suffering. The method of isolation and culture of PVSMCs is as following our previous description with some modifications [18]. The distal (4th generations) pulmonary veins were dissected from the lungs of Sprague Dawley (SD) rats (male, ages 8–10 weeks). The adventitia was carefully stripped off with forceps, and the endothelium was denuded by gently rubbing the luminal surface with a cotton swab. The isolated vessels was sequentially incubated for 40 minutes in ice-cold Hank's Balanced Salt Solution (HBSS, PH 7.2), 20 minutes in reduced-Ca^2+^ HBSS (20 µM CaCl_2_) at room temperature, and 23 minutes in reduced-Ca^2+^ HBSS containing collagenase, papain, Bovine Serum Albumin (BSA), and dithiothreitol at 37°C to disperse PVSMCs into a single-cell suspension. HBSS solution was then added into digestive solution and the tube was centrifuged for 5 minutes at 300 g. The supernatant was poured out and the cells pellet were washed with HBSS and centrifuged for 5 minutes at 300 g again. Then the cells were resuspended in Smooth Muscle Basal Medium (SMBM, Clonetics, Walkersville, MD) which contained 0.3% serum for 2 days, and then cultured in Smooth Muscle Growth Medium-2 (SMGM-2, Clonetics, Walkersville, MD) which contained 5% serum for 5–6 days in incubator with humid atmosphere of 5% CO_2_ -95% air at 37°C. Cells were growth arrested in Basal Smooth Muscle Medium with 0.3% serum for 12–24 hours before treatment.

### Hemodynamic measurements and lung histochemistry

Right ventricular pressure and right ventricular hypertrophy were measured using the method we described previously [19]. Briefly, a 23-gauge needle filled with heparinized saline was connected to a pressure transducer, and inserted via the diaphragm into the right ventricle (RV). The right ventricular systolic pressure (RVSP) and mean pulmonary arterial pressure (mPAP) were then measured. Right ventricular hypertrophy was evaluated as the wet weight ratio of right ventricle (RV) to left ventricle (LV) plus septum (S) and RV to body weight. To make intrapulmonary vessels visual, H&E staining was used on formalin-fixed and paraffin-embedded lung cross sections (5 µm). Pulmonary vascular parameters in H&E staining slides were measured with Image-Pro Plus 6.0 software by two independent operators in a masked manner. For immunofluorescence staining, we used the primary monoclonal antibodies raised against smooth muscle α-actin (Sigma), FITC-conjugated (excitation_λ = 488 nm) and CY3-conjugated secondary antibodies (excitation_λ = 543 nm). Slides were observed under a laser-scanning confocal fluorescence microscope (Nikon, Japan).

### Smooth muscle actin immunocytochemistry of PVSMCs

The method of PVSMCs immunostaining was performed as previously described. In a word, cells grown on 25 mm coverslips were serum free starved for 12–24 hours, fixed with ethanol, and then incubated with mouse monoclonal antibody for smooth muscle α-actin (α-actin; Sigma). The cells were then probed with Cy3-linked goat anti-mouse secondary antibody (Jackson Labs, West Grove, PA). Nucleuses were stained with YO-PRO-1 dimeric cyanine dye (Molecular Probes, Eugene, OR). The coverslips were then fixed on slides with FluoroGuard Antifade (Bio-Rad, Hercules, CA). Cells were examined under a laser-scanning confocal microscope with a Zeiss Plan-Neofluor 40 oil-immersion objective (Atlanta, GA).

### Exposure of rats to Chronic Hypoxia

Rats were randomized into a chronic hypoxic group and a normoxic control group. Rats in the chronic hypoxia group were exposed to hypoxic chamber for up to 21 days. The chamber was continuously flushed with a mixture of room air and N_2_ to maintain 10% O_2_. The successful development of the CHPH model was evaluated by measuring the weight ratio of the RV to the LV plus the septum [RV/(LV+S)], right ventricular systolic pressure (RVSP) and Hematocrit value.

### RNA extraction and real-time quantitative PCR

The total RNA of rat distal PV tissue and PVSMCs was extracted by using TRIzol reagent (Invitrogen, Carlsbad, CA) and RNeasy kit (Qiagen, Valencia, CA) as previously described [20]. RNA purification was determined by measuring ratios of A260/A280 and the value within the range of 1.8–2.0 was considered satisfactory for purity standards. cDNA was then synthesised by reverse transcription from 1000 ng total RNA and quantified by real-time quantitative PCR in an iCyclerIQ machine (BioRad) using Quanti-Tect SYBR Green PCR Master Mix (Qiagen). The qPCR reaction mixture contained 400 nM forward, reverse primers and a cDNA template. Primer sequences were as follows (5′-3′) TRPC1(F:AGCCTCTTGACAAACGAGGA, R:ACCTGACATCTGTCCGAACC);TRPC6(F:TACTGGTGTGCTCCTTGCAG,R:GAGCTTGGTGCCTTCAAATC);18S(F:GCAATTATTCCCCATGAACG;R:GGCCTCACTAAACCATCCAA). The procedure of real-time PCR consisted of three steps including a hot start at 95°C for 15 minutes, 40 cycles with each containing 94°C for 15 seconds, 57.5°C for 20 seconds, and 72°C for 20 seconds, and melting curves performed at 95°C for 1 minute, 55°C for 1 minute, and 80 repeats of increments of 0.5°C. Detection threshold cycle (CT) values were measured by iCyclerIQ software. Relative concentration of each transcript was calculated by using classic the Phaffl method. Data were presented as a ratio of TRPC to 18S in the same sample.

### Western blotting

Samples of PV tissue or PVSMCs were homogenized or lysed in buffer containing 62.5 mM Tris·HCl (pH 6.8), 2% SDS, 10% glycerol, 5% protease inhibitor cocktail, 1 mM EDTA, and 200 M 4-(2-aminoethyl) benzenesulfonyl fluoride hydrochloride. Protein concentration was measured by using the BCA Protein Assay Kit (Bio-Rad) with BSA as a standard (Calbiochem, San Diego, CA). The homogenized proteins were mixed with 150 mM dithiothreitol, heated at 95°C for 3 minutes, and resolved by 10% SDS-PAGE. These separated proteins were then transferred to polyvinylidene difluoride membranes (pore size 0.45 µm, BioRad) and incubated with rabbit polyclonal antibodies against TRPC1 (Sigma), TRPC6 (Sigma), STIM1 (Cell Signaling) or Orai1 (Alomone labs) for overnight or mouse monoclonal antibody that was specific for α-actin (Sigma) for 1 hour. Secondary antibodies were probed with horseradish peroxidase-conjugated goat anti-rabbit or anti-mouse IgG (KPL, Gaithersburg, MD) for 1 hour and then detected using an enhanced chemical luminescence system (GE Healthcare, Piscat away, NJ).

### Measurement of intracellular Ca^2+^ concentration

Ca^2+^ measurement experiments were accomplished as previously described [21]. PVSMCs were cultured on Coverslips which were loaded with 7.5 µM fura-2 AM (Molecular Probes, Eugene, OR) for 1 hour before calcium measurement experiments. Coverslips were then fixed in a specific polycarbonate chamber clamped in a heated platform (PH-2; Warner Instrument, Hamden, CT) on the stage of a Nikon TSE 100 inverted microscope (Nikon, Melville, NY). The chamber was perfused at 0.8–1 ml/minute with Krebs-Ringer bicarbonate solution (KRBS) containing: 118 mM NaCl, 4.7 mM KCl, 2.5 mM CaCl_2_, 0.57 mM MgSO_4_, 1.18 mM KH_2_PO_4_, 25 mM NaHCO_3_ and 10 mM glucose. During the experiments, temperature of coverslip was kept at 37°C and the solution was continuously equilibrated with 16% O_2_ and 5% CO_2_. The tubing and a manifold to an inline heat exchanger (SF-28, Warner Instrument) were used to warm the perfusate again just before it entered the polycarbonate chamber. The chamber platform was controlled at 37°C by a dual-channel heater controller (TC-344B, Warner Instrument). Data were collected by using InCyte II software (Intracellular Imaging, Cincinnati, OH). Intracellular Ca^2+^ concentration was estimated from the ratio of fura-2 fluorescence emitted at 510 nm after excitation at 340 nm to that after excitation at 380 nm (F340/F380). For measurement of VDCC, the concentration KCL was increased to 60 mM, and NaCl was decreased to 62.7 mM.

### Assessment of SOCE

Ca^2+^ restoration and Mn^2+^ quenching were used to assess SOCE. As described above, coverslips with PVSMCs growing on were perfused for 10–15 min with Ca^2+^-free KRB solution containing 5 µM nifedipine, 10 µM Cyclopiazonic acid (CPA), and 1 mM EGTA. First, we measured the basal intracellular Ca^2+^ concentration at 0.2 minute intervals and SOCE was measured from the increase in intracellular Ca^2+^ that was caused by restoration of extracellular Ca^2+^. Second, we measured fura-2 fluorescence excited at 360 nm at 0.5 minute intervals before and after the addition of MnCl_2_ (200 M) which act as Ca^2+^ surrogate and reduces Fura-2 fluorescence on binding to the dye to the perfusate. Since fluorescence excited at 360 nm was the same for Ca^2+^-bound and Ca^2+^-free fura-2, changes in fluorescence can be thought to be caused by Mn^2+^ alone. SOCE was evaluated by the rate at which fura-2 fluorescence was quenched by Mn^2+^.

### PVSMCs Proliferation assay

The proliferation assay of PVSMCs was performed by the Cell Proliferation Biotrak ELISA Kit (GE Healthcare), according to the operation manual. In short PVSMCs were seeded in 96 well plates in SMBM (Clonetics, Walkersville, MD) at a density of 4×10^3^ cells/well, cultured under normoxia or hypoxia (4% O_2_) condition for 60 hours and labeled with Brdu for 24 hours. The cells were then fixed, blocked, probed with anti-Brdu, developed with TMB substrate, and stopped with sulphuric acid. The optical density of wells was measured using a microplate reader (Bio-Rad) at 450 nm.

### PVSMCs migration assay

As we reported before [20], the migration of PVSMCs was assessed by using polycarbonate Transwell inserts (Membrane pore size 8 µm; Corning Incorporated, Lowell, MA). Briefly, PVSMCs were trypsinized and plated onto the membrane (1×10^5^ cells/insert) and incubated under normoxia and hypoxia (4% O_2_) condition for 24 hours. Cells were then fixed using 95% freezing-cold ethanol for 10 minutes, stained with Brilliant blue R Staining Solution for 5 minutes, and washed with PBS for three times. After staining, these cells were imaged under microscope at five fixed positions of the Transwell membrane and counted to represent the total cell number. The cells on the upper surface of the membrane inserts were then gently wiped off by using cotton swabs. Images for the migrated on the lower surface of membrane were taken at the same fields as indicated above for all PASMCs. Cell migration rates were measured as the counts ratio of the migrated cells to the total cells on each membrane.

### RNA interference

Small interfering RNA (siRNA) targeted to STIM1 (siSTIM1) and Orai1 (siOrail1) were designed and synthesized by Dharmacon (accession numbers XM_341896; siGENOME SMARTpool; Dharmacon, Lafayette, CO) and Shanghai GenePharma respectively. Primary cultured cells at 50–70% confluence were transfected with siSTIM1 or siOrai1, and nontargeting control siRNA using transfection vehicle (GeneSilencer; Genlantis, San Diego, CA) as carrier for 4 hours in serum-free SMBM in 5% CO_2_ at 37°C. The final concentration of each siRNA was 1000 ng/ml.

### Materials and drugs

Unless otherwise specified, all materials and drugs were obtained from Sigma. Fura-2 AM (Invitrogen) was prepared before the experiment as a 2.5 mM stock solution in 20% DMSO containing 20% pluronic F-127 (Invitrogen). Stock solutions of nifedipine and CPA were both made in DMSO at 30 mM.

### Statistical analysis

Data is represented as means ± SE; n is the number of experiments, which equals the number of animals providing veins or PVSMCs. When fura-2 fluorescence was measured for calculating intracellular Ca^2+^ concentration, the number of cells in each experiment was from 25 to 30. Statistical analyses were performed using variance (ANOVA) or Student's t-test. When *P*<0.05, comparison differences were considered significant.

## Results

### Characteristics of rat distal PVSMCs in primary culture

**Figure 1:**
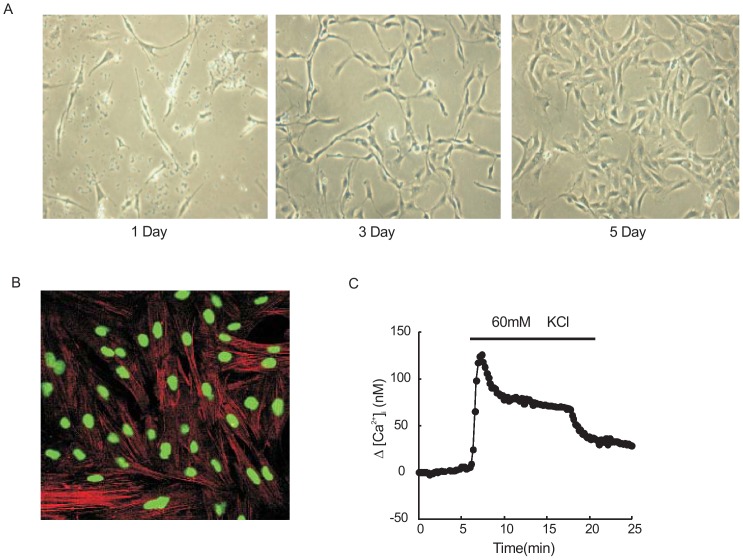
Characteristics of rat distal PVSMCs in primary culture. **A**: Representative phase-contrast microscopy images (objective ×20) of rat distal pulmonary vein smooth muscle cells (PVSMCs) cultured in growth media for 1, 3 and 5 days. **B**: α-actin fluorescent immunostaining in red, with nuclei counterstained in green in PVSMCs cultured for 3–4 days (objective ×40). **C**: Representative traces of time course of intracellular Ca^2+^ concentration responses to KCl (60 mM) in rat distal PVSMCs.

The primary cultured PVSMCs from rat distal pulmonary vein smooth muscle layer exhibited characteristics of smooth muscle cells as shown by the spindle-shaped appearance, expression of α-actin and presence of VDCC. Isolated primary PVSMCs were initially plated down in SMBM for 2 days and then cultured in SMGM-2 for 5–6 days. Observed with microscope, cells cultured in the growth medium were scrambled at the beginning, became scattered after two days, and showed well confluent and spindle-shaped on Day 5 (Figure 1A). Immunostaining for α-smooth muscle actin specifically in red showed typical elongated and cable-like fibers along the long axis of the cells (>95%). Nucleus were stained in green (Figure 1B). To confirm the presence of VDCC, which is another characteristic of smooth muscle cells, we measured the effect of 60 mM KCl on [Ca^2+^]_i_ in the cultured PVSMCs and all cells in visual fields exhibited clear increased [Ca^2+^]_i_ (Figure 1C). All these characteristics mentioned above indicated that these cells were smooth muscle cells origin.

### Basal [Ca^2+^]_i_ is increased in both freshly dissociated PVSMCs from CH exposed rats and *in vitro* cultured distal PVSMCs exposed to prolonged hypoxia

**Figure 2:**
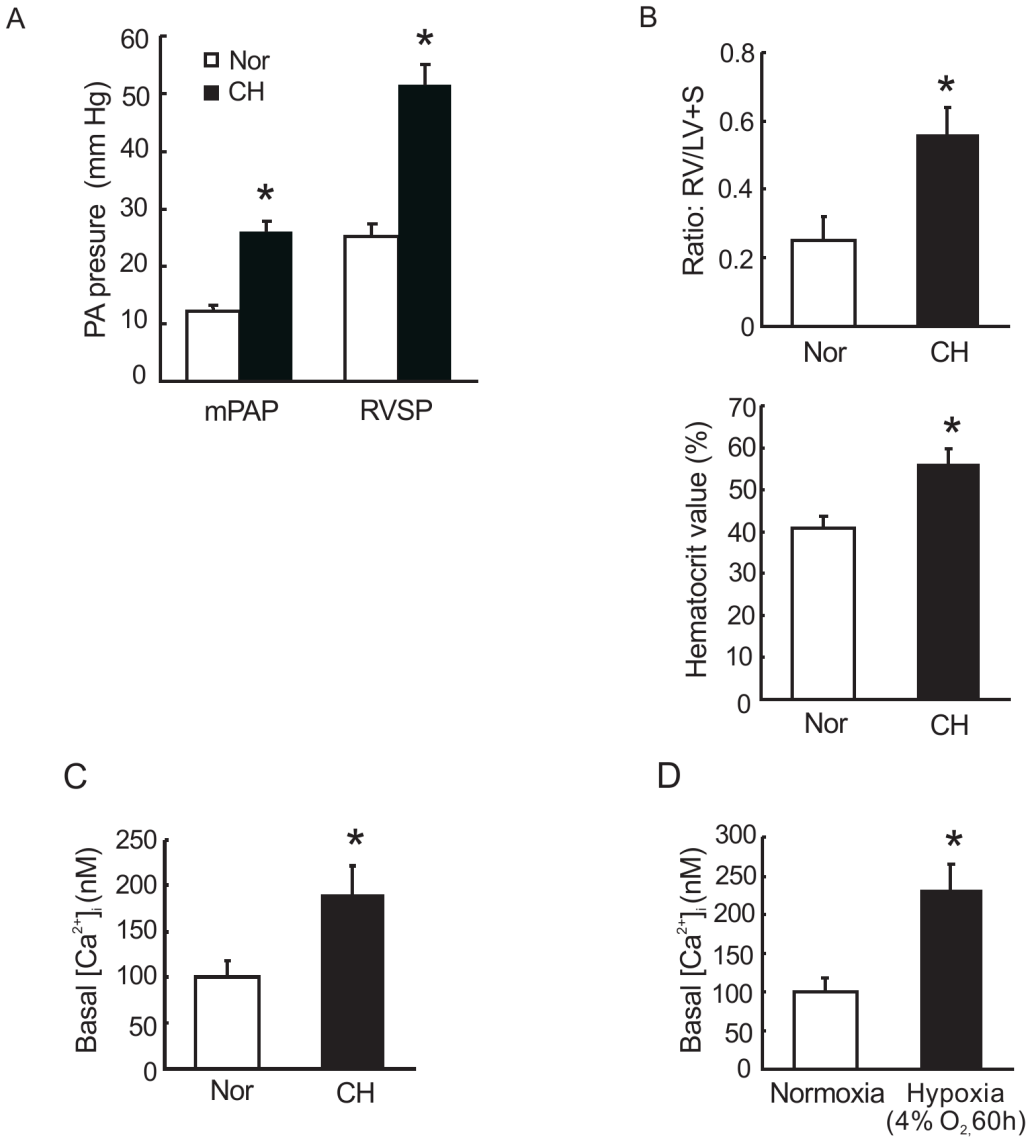
Effects of chronic hypoxia on right heart and pulmonary artery pressures and intracellular Ca^2+^ levels in PVSMCs. **A** and **B**: Parameters of chronically hypoxia pulmonary hypertension (CHPH) model. Animals were exposed to normoxia or hypoxia (10% O_2_) for 21 days. Bar graphs showing mPAP, RVSP, [RV/(LV+S)] and Hematocrit value respectively. Bar values are means ± SEM (n = 4 in each group). **C**: Basal intracellular Ca^2+^ concentration in fresh isolated PVSMCs from rats exposed to chronic hypoxia (10% O_2_, 21 days). Bar values are means ±SEM (n = 4 in each group). *P<0.05 versus normoxia control. **D**: Basal intracellular Ca^2+^ concentration ([Ca^2+^]_i_) in cultured PVSMCs from normoxia rats incubated under normoxia and hypoxia (4%O_2_, 60 hours). Bar values are means ± SEM (n = 4 in each group). *P<0.05 versus normoxia control.

Rats exposed to chronic hypoxia (10% O_2_ for 21 days) successfully developed CHPH. As shown in Fig. 2A, mean pulmonary artery pressure (mPAP) increased from 12.3±0.42 mmHg in normoxic control rats to 26.2±0.77 mmHg in hypoxic rats, and right ventricular systolic pressure (RVSP) increased from 25.1±0.98 mmHg in normoxic control rats to 51.4±1.65 mmHg in hypoxic rats. This effect of CH was accompanied by right ventricular hypertrophy (RVH), which was indicated by increases in the ratio of right ventricle to left ventricle plus septum weight [RV/(LV+S)] and Hematocrit value (%). As shown in Figure 2B, the ratio of RV/(LV+S) was increased from 0.25±0.07 in normoxic controls to 0.56±0.8 in hypoxic rats and the Hematocrit value (%) was increased from 41±2.81 in normoxic to 65±3.82 in hypoxic rats.

Distal PVSMCs from CHPH rats (10% O_2_, 21 days) and nomoxic control rats were prepared for basal [Ca^2+^]_i_ measurement, respectively. Cells from CHPH rats showed significantly increased basal [Ca^2+^]_i_ (190±31 nM) compared with that from controls (100±17 nM) as shown in Figure 2C. In order to confirm this observation, distal PVSMCs were isolated from normal rats and exposed to prolonged hypoxia (4% O_2_, 60 hours) or normoxia. Basal [Ca^2+^]_i_ in the hypoxic PVSMCs (232±34 nM, n = 4) was significantly greater than that in nomoxic cells (100±18 nM, n = 4, *p*<0.05; Figure 2D).

### Hypoxia elevated SOCE of PVSMCs both *in vivo* and *in vitro*

**Figure 3:**
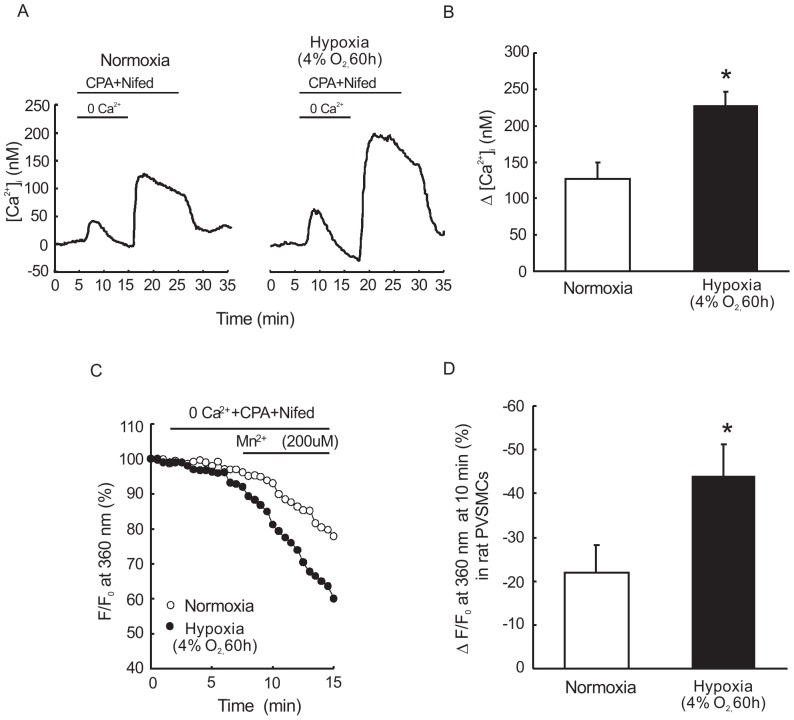
Hypoxia increased basal [Ca^2+^]_i_ of PVSMCs *in vivo* and *in vitro*. **A**: Time course effects of hypoxia on basal [Ca^2+^]_i_ change before and after restoration of extracellular Ca^2+^ to 2.5 mM in distal PVSMCs perfused with Ca^2+^-free Kreb solution containing 10 µM CPA, 1 mM EGTA and 5 µM nifedipine. **B**: Average peak change in intracellular Ca^2+^ concentration and after restoration of extracellular Ca^2+^ in cells exposed to hypoxia or normoxia control. *P<0.05 versus normoxia control. **C**: Time course of fura-2 fluorescence at 360 nm normalized to values before and after administration of MnCl_2_ (200 µM) to distal PVSMCs perfused with Ca^2+^-free KRB solution containing 10 µM CPA, 1 mM EGTA, and 5 µM nifedipine. **D**: Average decrease in fura-2 fluorescence at 10 min after administration of MnCl_2_ to cells *P<0.05 versus normoxia control.

We assessed SOCE in two ways. By measuring both intracellular Ca^2+^ response to extracellular Ca^2+^ restoration and Mn^2+^ quenching of fura-2 fluorescence after store depletion with CPA, we found that hypoxia can enhance SOCE. As seen in Figure 3A and 3B, we measured the peak increase in [Ca^2+^]_i_, caused by restoration of extracellular [Ca^2+^] in PVSMCs perfused with Ca^2+^-free Krebs solution containing 10 µM CPA and 5 µM specific L-type VDCC blocker nifedipine, indicating a calcium release from endoplasmic reticulum. Subsequently, restoration of extracellular Ca^2+^ caused a second larger peak increase of [Ca^2+^]_i_ which was significantly greater in the hypoxia exposed PVSMCs (228.1±19 nM, n = 4 experiments in 85 cells) than that observed in normoxic control cells (127.2±23 nM, n = 4 experiments in 96 cells), indicating enhanced SOCE in the hypoxic PVSMCs.

Considering that the increase in [Ca^2+^]_i_ caused by restoration of extracellular Ca^2+^ can be affected by factors other than SOCE such as changes in membrane potential, we also measured the rate at which Mn^2+^ quenched Fura-2 fluorescence. As shown in Figure 2C and 2D, in the presence of CPA and nifedipine, Fura-2 fluorescence excited at 360 nm was decreased along time by perfusing PVSMCs with Ca^2+^-free Krebs containing 200 µM Mn^2+^. Mn^2+^ quenching, expressed as the percentage decrease in F360 at 10 min in hypoxia exposed PVSMCs at 40.1±7.1% (n = 4 experiments in 95 cells) was significantly enhanced compared with 22.1±6.1% (n = 4 experiments in 108 cells) in normoxia control cells. It suggests that extracellular Ca^2+^ entry through store-operated Ca^2+^ channels (SOCCs) in hypoxic PVSMCs is enhanced when stores are depleted.

### TRPC6 expression is increased in PVSMCs exposed to prolonged hypoxia and PV tissue from hypoxic rats

**Figure 4:**
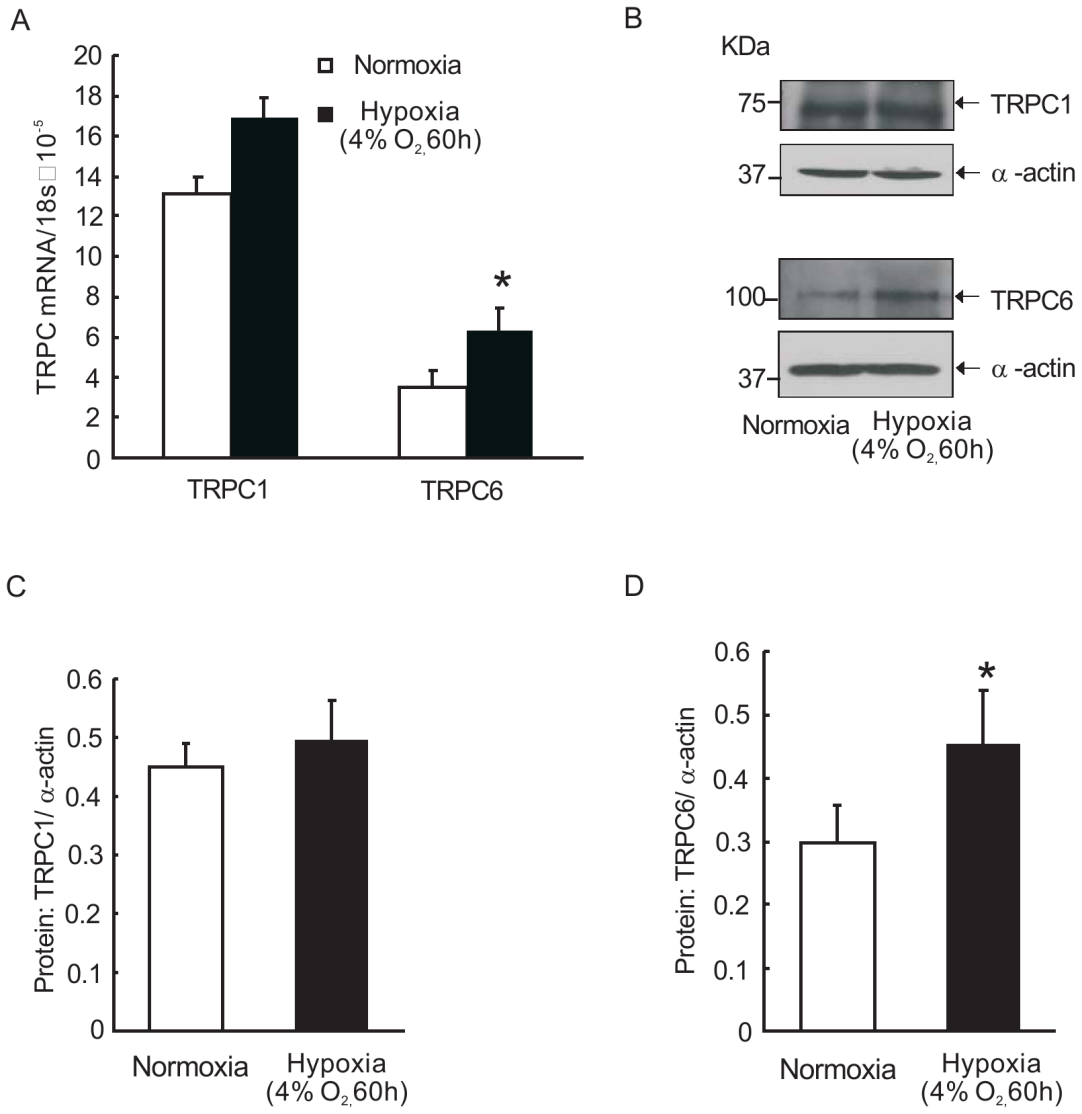
Prolonged hypoxia up-regulated transient receptor potential 6 (TRPC6) expression *in vitro*. **A**: TRPC1 and TRPC6 mRNA relative to 18s determined by real-time quantitative PCR. **B** and **C**: TRPC1 proteins determined by Western blotting. Representative blots (B) and mean intensity (C) for TRPC1 blots relative to α-actin. **D** and **E**: TRPC6 proteins determined by Western blotting. Representative blots (D) and mean intensity (E) for TRPC1 blots relative to α-actin. Bar values are mean ± SEM (n = 3 in each group). *P<0.05 versus respective normoxia control.

To investigate whether hypoxia alters TRPCs expression in PVSMCs, distal PVSMCs were isolated from normal rats and were cultured under hypoxic condition (4% O_2_, 60 hours) and normoxic condition as control. Expression of TRPCs in PVSMCs was detected and the results showed that TRPC6, but not TRPC1, was significantly increased at both mRNA and protein levels in hypoxic PVSMCs, compared with that in normoxic controls (Figure 4A–E).

**Figure 5:**
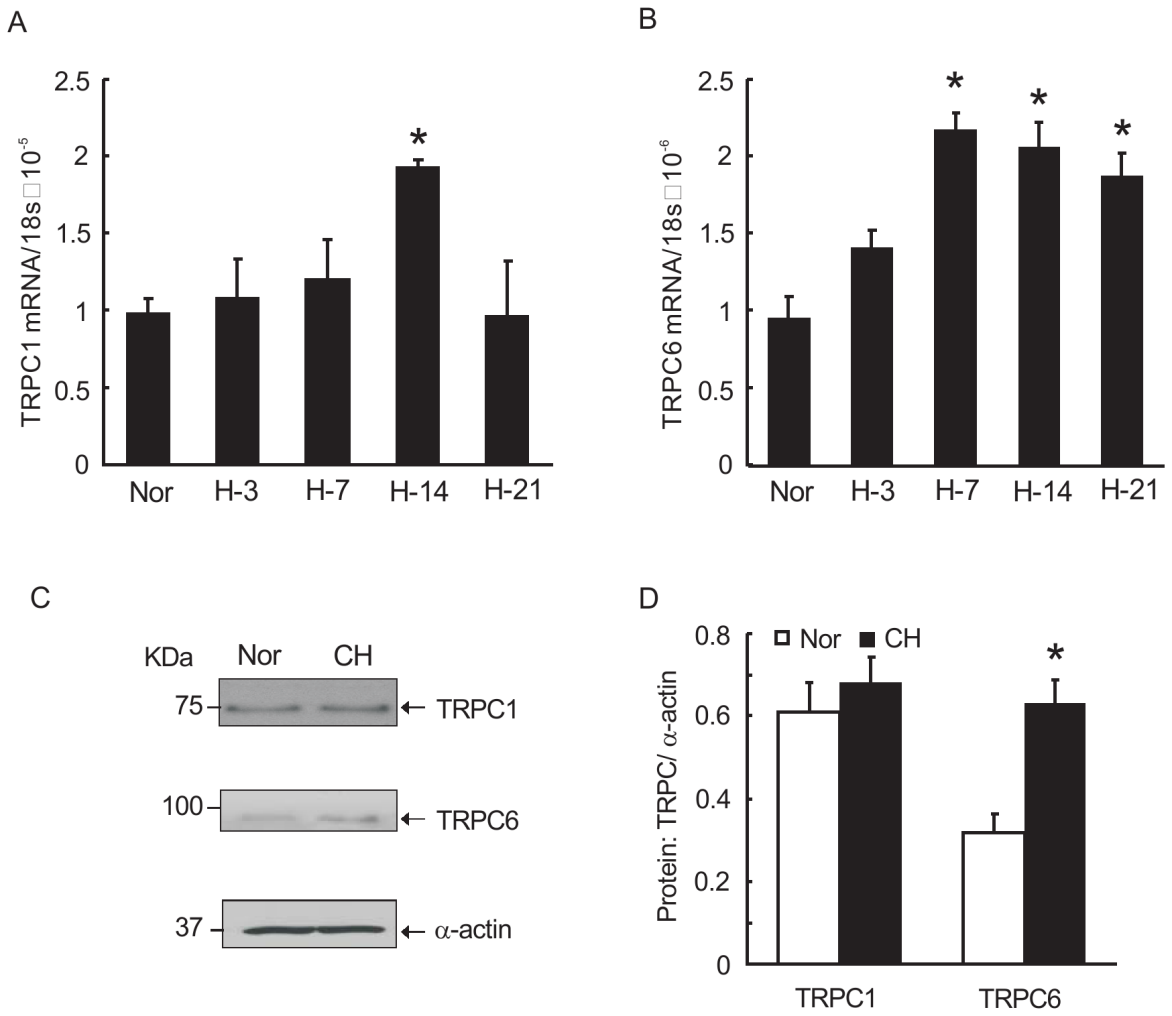
Chronic hypoxia increased TRPC6 expression in PV from hypoxia exposed rats. TRPC expression in distal PV tissue from rats exposed to normoxia or hypoxia (10% O_2_, 21 days). **A** and **B**: the mRNA expression of TRPC1 and TRPC6 in distal PV from rats exposed to hypoxia (10% O_2_) for different days compared with that in control group. **C** and **D**: TRPC1 and TRPC6 proteins expression determined by Western blotting. Representative blots (C) and mean intensity (D) for TRPC1 and TRPC6 blots relative to a-actin. Bar values are mean±SEM (n = 3 in each group). *P<0.05 versus respective normoxia control.

To confirm this observation, we further detected the mRNA and protein expression of TRPC1 and TRPC6 in distal PV tissues from rats exposed to hypoxia for 3, 7, 14 and 21 days. The expression of TRPC1 mRNA in rat PV tissue was barely changed, with only a transient and mild increase under 14 days of hypoxia exposure (Figure 5A). While TRPC6 mRNA significantly increased when exposed to hypoxia for 7 days and remained statistical difference until 21 days after hypoxia exposure (Figure 5B). Moreover, expression of TRPC1 and TRPC6 protein in distal PV tissues from hypoxic rats (10% O_2_, 21 days) and normoxic controls was detected by western blotting. Consistent with the results from PVSMCs, expression of TRPC6, but not TRPC1, in PV from hypoxic rats was significantly increased compared with that from normoxic controls (Figure 5C, D).

### Hypoxia increases proliferation and migration of rat distal PVSMCs

**Figure 6:**
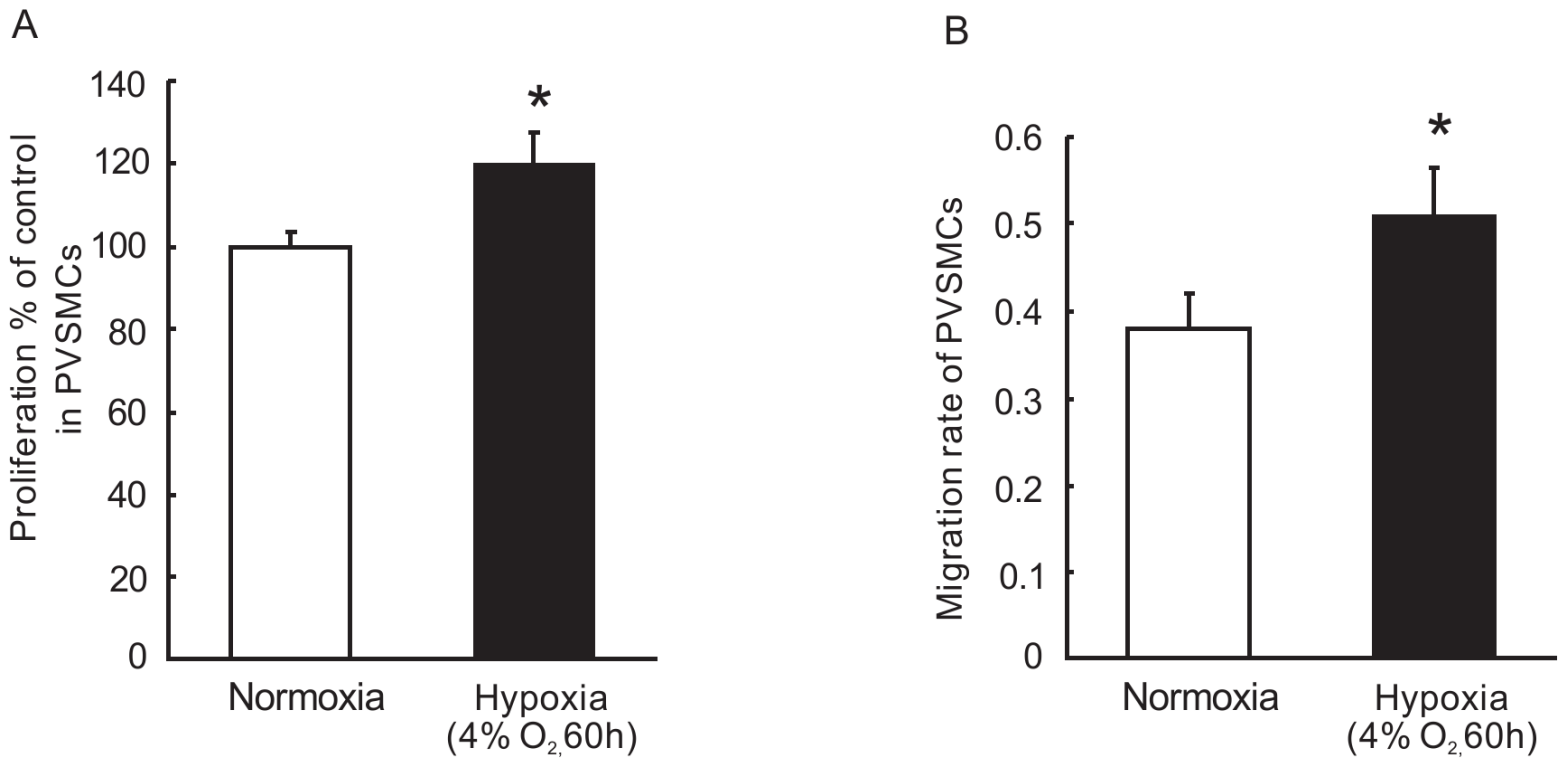
Hypoxia increased proliferation and migration of PVSMCs. A: PVSMCs were incubated under normoxia or hypoxia condition (4% O_2_, 60 hours). Cells proliferation was assessed by analyzing the BrdU incorporation rate under normoxia or hypoxic conditions. Values were normalized to normoxia alone and presented as percentages. B: PVSMCs were incubated under normoxia or hypoxia condition (4% O_2_, 24 hours). The migration rates of PVSMCs were determined by calculating the ratios of migrated cells (on the lower surface of trans-well membrane) to the total cells (cells on both sides of trans-well membrane). *P<0.05 versus respective normoxia control. (n = 4 in each treatment group).

Rat distal PVSMCs were isolated and exposed to prolonged hypoxia (4% O_2_, 60 hours). Cells exposed to normoxia served as controls. As seen in Figure 6, prolonged hypoxia enhanced both proliferation and migration of PVSMCs. The proliferation of PVSMCs increased from about 100±4.2% in normoxic cells to 120.4±7.4% in those exposed to prolonged hypoxia (P<0.05, Figure 6A). The migration rate increased from 0.38±0.04 in normoxic cells to 0.51±0.06 in PVSMCs exposed to prolonged hypoxia (4% O_2_, 24 hours) (P<0.05, Figure 6B).

### STIM1 knockdown reduced SOCE in cultured rat distal PVSMCs

**Figure 7:**
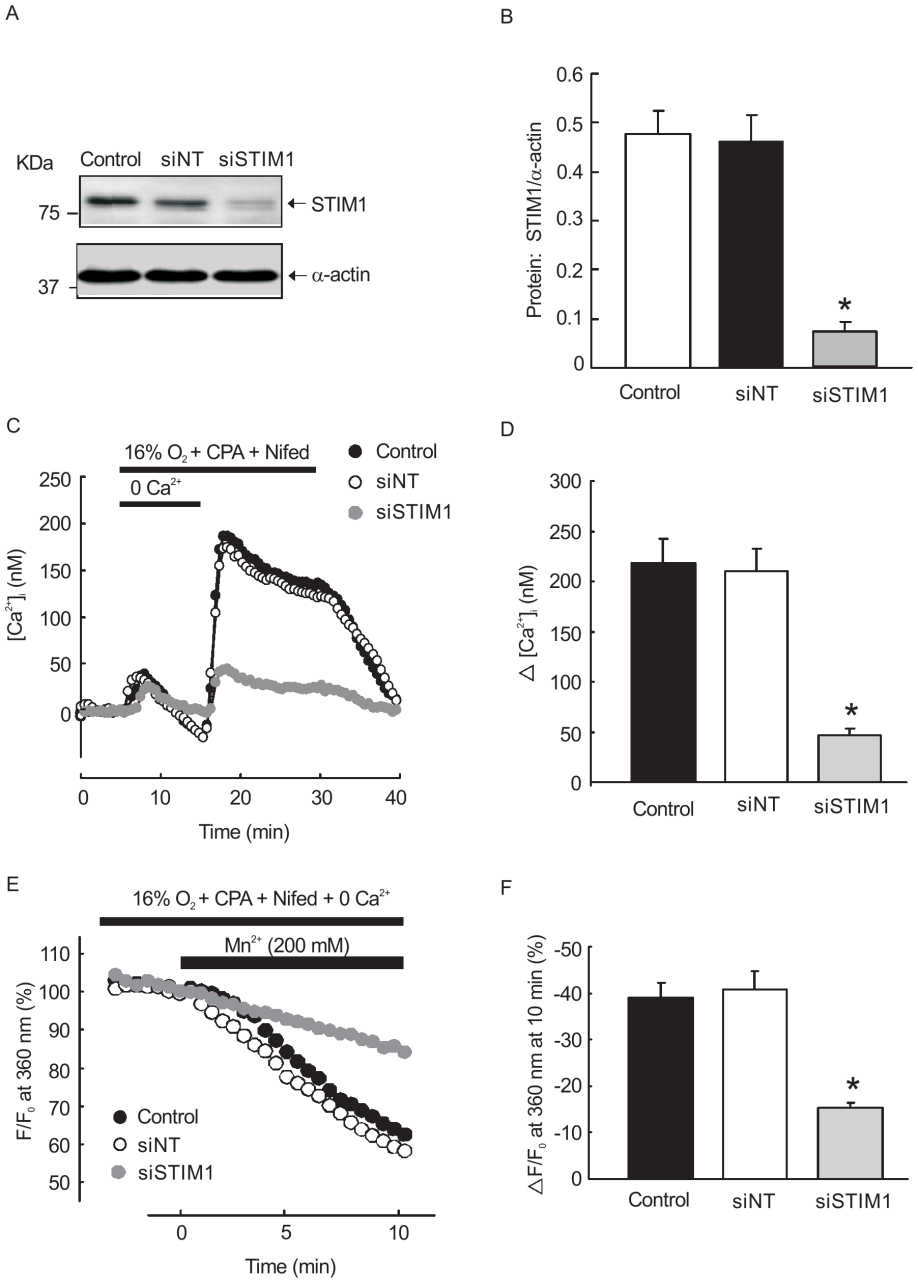
Effects of siSTIM1 transfection on basal [Ca^2+^]_i_ and SOCE in rat PVSMCs. **A, B**: Western blot showing expression of STIM1 and α-actin protein in rat PVSMC treated with siNT, siSTIM1 and transfection vehicle alone (control). **C, D**: [Ca^2+^]_i_ change (Δ[Ca^2+^]_i_) before and after restoration of extracellular [Ca^2+^]_i_ to 2.5 mM in PASMC perfused with Ca^2+^-free Krebs ringer bicarbonate (KRB) solution in each group. **E, F**: The changes of SOCE in siSTIM1, NTsiRNA transfected PVSMCs and transfection vehicle alone treated control. *P<0.05 versus siNT control. (n = 3 in each treatment group).

Specific siRNA against STIM1 (siSTIM1) was synthesized and non-targeted siRNA (siNT) was used as a control. PVSMCs were were incubated with siSTIM1 or NT siRNA, control cells were treated with transfection vehicle alone. The specificity and knockdown efficiency of siSTIM1 was detected using Western blotting. As shown in Fig. 7 A and B, siSTIM1 effectively reduced STIM1 protein expression by 81±5.6% (n = 4, *P*<0.01). Changes in the [Ca^2+^]_i_ (Δ[Ca^2+^]_i_) and SOCE were measured in each respective treated cell. Results showed that the Δ[Ca^2+^]_i_ was significantly reduced in STIM1 knockdown group compared with that in NT siRNA and control groups (n = 4 experiments in 112 cells, *P*<0.01). There was no significant difference in Δ[Ca^2+^]_i_ between NT siRNA treated and control cells (Figure 7 C and D). Consistent with the results of Δ[Ca^2+^]_i_, Mn^2+^ quenching measurement revealed that siSTIM1 transfection significantly attenuated the Mn^2+^ quenching compared with NT siRNA treated group and control group n = 4 experiment in 108 cells, *P*<0.01) (Figure 7 E and F).

### Orail1 knockdown reduced SOCE in cultured rat PVSMCs

**Figure 8:**
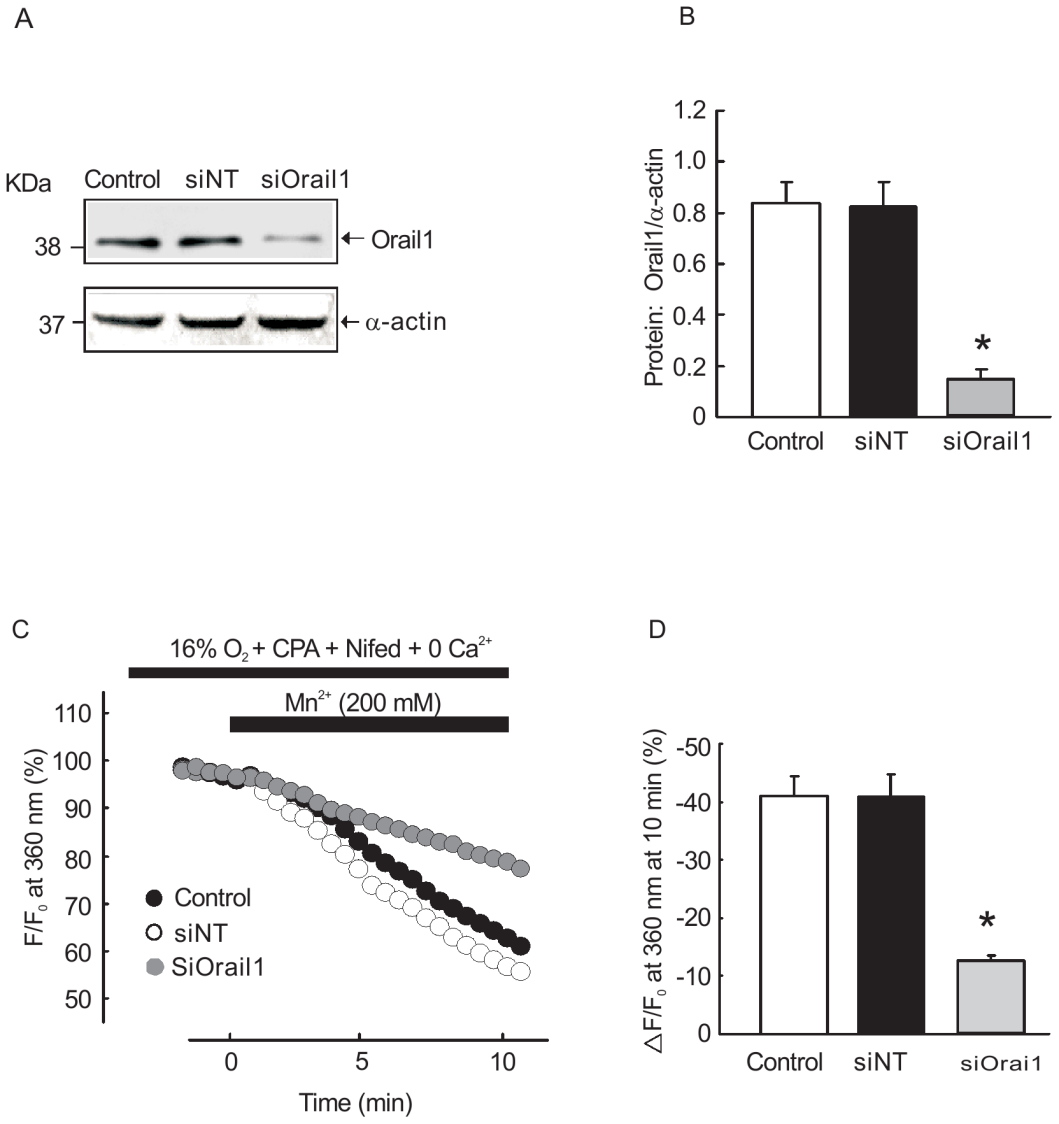
Effects of siOrai1 transfection on basal [Ca^2+^]_i_ and SOCE in rat PVSMCs. **A, B**: Western blot showing expression of Orai1 and α-actin protein in rat PVSMC treated with siNT, siOrai1 and transfection vehicle alone (control). **E, F**: The changes of SOCE in siOrai1, NTsiRNA transfected PVSMCs and transfection vehicle alone treated control. *P<0.05 versus siNT control. (n = 3 in each treatment group).

Similarly, specific siRNA against Orai1 (siOrai1) was synthesized and non-targeted siRNA (siNT) was used as a control. Cultured PVSMCs were incubated with siSTIM1 or NT siRNA, control cells were treated with transfection vehicle alone. The specificity and knockdown efficiency of siOrai1 was detected using Western blotting. As shown in Fig. 8 A and B, siOrai1 effectively reduced Orai1 protein expression by 87±2.6% (n = 4, *P*<0.01). Additionally, SOCE was also measured using Mn^2+^ quenching measurement, which revealed that siOrai1 transfection significantly decreased the Mn^2+^ quenching compared with NT siRNA treated group (n = 4, *P*<0.01) (Figure 8C and D).

## Discussion

In this study, we investigated the effect of hypoxia on SOCE and TRPCs expression in rat distal pulmonary vein smooth muscle. The proliferation and migration of rat distal PVSMCs exposed to hypoxia were also detected. Our results revealed that hypoxia increased SOCE and enhanced TRPC6 expression in rat distal pulmonary vein smooth muscle. Experiments with rat distal PVSMCs *in vitro* showed that hypoxia increased proliferation and migration of PVSMCs. Rat distal pulmonary vein smooth muscle was demonstrated to exhibit a similar response to hypoxia in compare to pulmonary artery smooth muscle.

Previous studies of mechanisms underlying CHPH development mainly focused on the effects of hypoxia on pulmonary arteries and PASMCs. It was found that pulmonary arterial vasoconstriction and structural remodeling were two major links during CHPH development. However, based on the pathophysiology of pulmonary circulation, when oxygen partial pressure in pulmonary circulation decreases, hypoxemia first affected pulmonary vein. Thus, exploring the mechanism underlying CHPH development from the PV side is necessary and meaningful. For investigating the function of pulmonary vein, sufficient and purified PVSMCs are required. In the past, we developed an efficient method to isolate and culture PVSMCs from rat distal pulmonary veins [18]. Since the main PV near the left atrium contain cardiomyocytes [22], we isolated distal PV (the 4th generation) to reduce the chance of contamination of cardiomyocytes. As shown, we got pure PVSMCs characterized by the morphological and function of vascular smooth muscle cells by using the method. Our PVSMCs specifically expressed alpha-smooth muscle actin and possessed the L-type VDCC, which indicated their vascular smooth muscle cell origin.

In the past decades, an increasing number of studies reported that similar to pulmonary artery, hypoxia can also induce remodeling in pulmonary vein [12], [23]. In this study, CHPH model was successfully developed based on our previous observation indexes. Considering the essential effect of enhanced intracellular Ca^2+^ concentration to hypoxia highlighted smooth muscle cell proliferation and migration, we found that both freshly isolated PVSMCs from CHPH rats and cultured PVSMCs exposed to prolonged hypoxia exhibited elevated intracellular Ca^2+^ concentration than cells from normoxic rats. These findings are similar to those observed in PA and PASMCs from different species in the past, suggesting that basal Ca^2+^ concentration increase in PV is pivotal during CHPH development.

Based on previous knowledge, the enhanced intracellular Ca^2+^ concentration can be achieved mainly by: the Ca^2+^ release from sarcoplasmic reticulum, Ca^2+^ influx from extracellular fluid through voltage-dependent calcium channels (VDCCs), receptor-operated Ca^2+^ channels (ROCCs), or SOCCs [24]. In our study, we used nifidepine, a specific blocker of VDCCs, to exclude the influence of Ca^2+^ influx via VDCCs. We calculated the SOCE as the increase in intracellular Ca^2+^ following the restoration of extracellular Ca^2+^ and as the decreased rate of Mn^2+^ quenching in PVSMCs. We found prolonged hypoxia could enhance the SOCE in PVSMCs, which was similar to that in PASMCs. SOCC is known to be mainly composed of the classical TRPC family members, among which TRPC1 and TRPC6 have been found to be expressed in the rat pulmonary vein and PVSMCs at both mRNA and protein levels [16]. In vasculature, TRPC4 is thought to be expressed more by endothelial cells rather than smooth muscle cells. Therefore, we did not detect TRPC4 in this study.

TRPC6 is usually considered to be a key component of ROCCs, which could be activated by diacylglycerol (DAG) and other signaling messengers involved in the G protein signal pathway [21] [25], [26]. However, more recent studies indicated that TRPC6 is an important component of SOCCs and contributes to cellular SOCE process [27], [28], although there is also study suggesting less relevance [29]. In previous study, the increase of TRPC6 expression was associated with the increased SOCE in proliferative PASMCs, whereas inhibition of TRPC6 expression with antisense oligonucleotides or bosentan could reduce SOCE that induced by sarcoplasmic reticulum Ca^2+^ store depletion and mitogen-mediated PASMCs proliferation [28], [30], [31]. Knockdown of TRPC6 by siRNA can attenuate SOCE and proliferation rate in human hepatoma cells [27]. Our previous study demonstrated that TRPC6 compose functional SOCCs in rat distal PASMCs, supported by that knockdown of TRPC6 expression by specific siRNA could reduce hypoxia-induced increases in SOCE [19]. All these evidence strongly demonstrated that TRPC6 participates in the formation of SOCCs and contributes SOCE. Based on what we mentioned above about the relevant relationship among TRPC6 expression, intracellular calcium homeostasis and the development of Pulmonary hypertension, it is reasonable to hypothesize that hypoxic calcium entry and cell proliferation in PV are potentially mediated via the TRPC–SOCE–[Ca^2+^]_i_ signaling pathway.

Unlike to TRPC6, TRPC1 is mostly determined as a primary component of SOCCs, which provides a pathway for SOCE, thus participating in the regulation of intracellular Ca^2+^ concentration in various cell types, including PASMCs [32], [33], [34]. However, in this study, we observed that increased TRPC6 expression, but not TRPC1, correlated with elevated [Ca^2+^]_i_, enhanced SOCE and triggered proliferation and migration in PVSMCs. These results suggest that although similar enhanced intracellular Ca^2+^ concentration takes place in both PA and PV, the detail mechanism seems different. These results could potentially explain why PV exhibits less degree of remodeling than PA, and why PVSMCs exhibit smaller increase in SOCE and basal [Ca^2+^]_i_ than PASMCs, when exposed to hypoxia. Moreover, STIM1 protein has been identified to act as Ca^2+^ sensor and is located in the internal Ca^2+^ stores. After emptying the stores STIM molecule aggregate to activate Orai channels which are responsible for SOCE. In PASMCs, it is well accepted that stimuli inducing ER depletion leads to STIM1 translocation to the plasma membrane, interacts with and activates Orai and TRPC channels to mediate SOCE. However, whether the similar machinery is also present in PVSMCs remains unknown. In this study, by using specific siRNA knockdown strategy, we performed additional experiments to demonstrate that like PASMCs, in PVSMCs, STIM1 and Orai1, also contribute to and largely mediate SOCE, respectively. Under this condition, in hypoxic PVSMCs, the increased TRPC6 expression is likely responsible for hypoxia-elevated intracellular calcium homeostasis.

It is well known that hypoxia induced pulmonary vascular remodeling is directly associated with the PA smooth muscle cell proliferation and migration [35], [36], during which the concentration of intracellular Ca^2+^ has been reported to be an important determinant [37], [38], [39]. In this study, given the fact of the increased [Ca^2+^]_i_ and SOCE in hypoxic PVSMCs, we further confirmed that hypoxia also elevated the proliferation and migration of PVSMCs, suggesting a similar pattern to that in hypoxic PASMCs.

In conclusion, we initially demonstrated that hypoxia induced enhanced SOCE in both *ex vivo* freshly isolated and *in vitro* cultured rat distal PVSMCs, leading to elevated basal [Ca^2+^]_i_ enhancement in PVSMC. This enhancement was mainly dependent on the increased expression of TRPC6. Hypoxia increased basal [Ca^2+^]_i_ triggered proliferation and migration of PVSMCs, and led to PV structural remodeling which contributed to pulmonary circulation peripheral resistance enhancement.
